# Laparoscopic splenectomy in patients with idiopathic thrombocytopenic purpura and very low platelet count

**DOI:** 10.22088/cjim.13.2.8

**Published:** 2022

**Authors:** Abbas Abdollahi, Seyed Maryam Naghibi, Hamed Shariat Razavi, Alireza Tavassoli, Azadeh Jabbari Nooghabi, Mehdi Jabbari Nooghabi

**Affiliations:** 1 Surgical Oncology Research Center, Mashhad University of Medical Sciences, Mashhad, Iran; 2 Endoscopic and Minimally Invasive Surgery Research Center, Mashhad University of Medical Sciences, Mashhad, Iran; 3 Department of Statistics, Ferdowsi University of Mashhad, Mashhad, Iran

**Keywords:** Laparoscopy, Splenectomy, Purpura, Thrombocytopenic, Idiopathic, Steroids

## Abstract

**Background:**

Chronic idiopathic thrombocytopenic purpura (ITP), is an autoimmune disease associated with a reduction in circulating blood platelets under 150±10^9^g/L which persists longer than 6 months without any specific cause. With the current study, we aimed to evaluate the efficacy and safety of laparoscopic splenectomy in ITP patients with a very low platelet count and normal coagulation status.

**Methods:**

From April 2007 to January 2012, laparoscopic splenectomy was performed on 60 patients with chronic ITP who could not achieve a sustained recovery after steroid therapy.

**Results:**

Patients consisted of male/female ratio of 24 (40%):36 (60%) with the mean age of 53±15.1 years. All patients had normal coagulation state even with very low platelet count (below 5×10^3^) before laparoscopic splenectomy. The mean operative time was 140.00±15.00 minutes. Blood transfusion was required in 10 (16.7%) and 8 (13.3%) patients before and after the operation, respectively. Preoperative transfusion of PRBC was not statistically significant between groups (P=0.265). Bleeding complications during within or after surgery was rare (5.0%). Convalescence was rapid and the mean hospital stay was 3.58±0.68 days (1-14 days) which shows that there was no significant difference in operative time and after operative hospitalization time among the three groups (P=0.070). The patients made a good uneventful recovery and were followed for at least one week who exhibited no postoperative problems.

**Conclusion:**

Laparoscopic splenectomy should be considered initially in the management of ITP. Also, very low platelet count should not be contraindicated for laparoscopic splenectomy in ITP patients and sometimes perioperative platelet transfusion may be unnecessary.

Idiopathic thrombocytopenic purpura (ITP) is characterized by a low platelet (Plt) count, which has been shown to be immune-mediated platelet destruction (1-5). Therefore, by clearing the organ which is responsible for the clearance of antibody-coated platelets, this problem will be resolved. Based on the autoimmune pathogenesis of ITP, glucocorticoids have been the standard initial treatment for the patients with moderate to severe thrombocytopenic purpura (6-8). Splenectomy, which is usually considered as the second line therapy for treatment of idiopathic thrombocytopenic purpura has been a well-recognized and effective therapeutic modality with a rate of 70–90% of hematologic response in patients either refractory to or dependent upon medical treatment (6-8).

Since early 1992, when the first application of laparoscopic procedures for splenectomy was introduced, several retrospective studies have confirmed that laparoscopic spleen (LS) is applied and confident, and it is related with lower morbidity and faster recovery time compared to the conventional surgery (9-13). Although low preoperative platelet count may result in bleeding complications, it is not absolutely contraindicated for LS. In some studies, low platelet count (<30×$10^{9}$109/L) is considered to be inefficacious in LS because of higher morbidity and more blood transfusion requirements (14).

Laparoscopic procedure is associated with lower postoperative pain, decreased length of hospitalization, and improved pulmonary function, which allows for rapid recovery and ultimately increases the universal acceptance (15-17). Several published studies have shown the technical feasibility of LS with low postoperative morbidity and mortality rates (12, 18, 19). With the current study, we aimed at evaluating the safety of laparoscopic splenectomy in patients with idiopathic thrombocytopenic purpura and very low platelet count.

## Methods

The present study is a prospective study of 60 patients with chronic idiopathic thrombocytopenic purpura (ITP) who underwent laparoscopic splenectomy between April 2007 and January 2012 at Ghaem Hospital of Mashhad, Iran. The study was confirmed by the Ethics Committee of Mashhad University of Medical Sciences. All patients in this study were determined as having ITP based on the guidelines of the American Society of Hematology (2). Inclusion criteria for laparoscopic splenectomy (LS) were chronic ITP patients who were refractory to corticosteroids, relapse of thrombocytopenia after tapering of steroid or development of side effects with high doses of steroids. According to the preoperative Plt count, the patients were divided into three groups: Group I with Plt count < 10×10^9^/L, group II with Plt count of 10-30×10^9 ^/L and group III with Plt count > 30×10^9^/L. All patients had normal coagulation profile. The clinical and operative data of all patients were collected.

**Operative technique: **The patients were given a polyvalent pneumococcal vaccine at least 2 weeks before surgery. Previous to the surgery, patients also received intravenous hydrocortisone (100 mg in three doses per day) for at least 5 days. After general anesthesia and endotracheal intubation, we used the standard surgical technique with the patients in the right lateral decubitus position and application of three or four trocars. All procedures were performed with the same surgeon and an assistant surgeon.

**Statistical analysis: **Data was analyzed using R statistical software (Version 3.2). The qualitative variables were analyzed using frequency and percentage in descriptive statistics and chi-square and the Fisher’s exact tests as an inferential statistic. Also, we used the mean and standard deviation to describe the numerical variables. Furthermore, independent sample t-test, Mann-Whitney test, one way ANOVA with LSD multiple comparison test and Kruskal-Wallis test were used to compare the numerical variables between groups.

## Results

**Patients Characteristics: **There were 24 (40.00%) men and 36 (60.00%) women, ranging in age from 14 to 76 years (mean age of 53.00±15.10 years). The time duration of disease from diagnosis to surgery was various. Most patients presented with mild to moderate mucosal bleeding, purpura, epistaxis, and menorrhagia, gastrointestinal and rarely central nervous system hemorrhage. Oral corticosteroids were administered for all patients before admission.

The mean Plt count on admission was (45.30±42.70) ×10^9^/L. We divided the patients into three main groups: 13 (21.67%) patients had a Plt count < 10 × 10^9^/L (group I) , 17 (28.33 %) patients with Plt count of 10-30 × 10^9^/L (group II) and the other 30 (50.00 %) patients had a Plt count >30 × 10^9^/L (group III). Blood transfusion was required in 10 (16.7%) and 8 (13.3%) patients before and after the operation, respectively. There was no platelet transfusion before the surgery. According to table 1, there were no significant differences between three groups in age, gender, and preoperative transfusion of packed red blood cell (PRBC) (p>0.05).

**Operative outcome: **The comparison of the operative outcome data (operative time, length of hospitalization and PRBC transfusion) of groups is shown in table 2. In 2 (3.33%) patients, accessory spleens were found intraoperatively. The overall mean time of general anesthesia was 2.38±0.78 hours (around 143 minutes in average). The mean length of postoperative hospitalization was 3.75±2.29 (range, 1-14) days. There was no significant difference in operative time and postoperative hospitalization time among the three groups (P=0.070). Eight (13.33%) patients experienced massive bleeding (more than 500 mL) during surgery because of splenic capsule tearing or from venous branches. Transfusion of PRBC was required for all these 8 patients after surgery; and in two (25%) patients, the procedure was converted to open surgery for control of bleeding. 

Preoperative transfusion of PRBC was not statistically significant between groups (P=0.265) (table 1). However, postoperative blood transfusion was higher in group I (3.60±1.14 packs) compared to group II (3.00±2.83 packs) and III (3.00±1.41 packs) but was not statistically significant (P=0.873) (table 2). To evaluate the efficacious factors in PRBC transfusion requirement, the patients were divided into two groups: those who did not receive PRBC transfusion (52, 86.67%) and those who received (8, 13.33%). The results are presented in table 3. According to (table 3), in patients who required blood transfusion, the length of hospitalization was higher than the other group (7.38±4.31days versus 3.19±1.07 days, P=0.001).

 Also, the comparison of the mean Plt count in the day of surgery, before and after surgery demonstrated a significant difference between the two groups. Other variables such as age, sex, preoperative hemoglobin (Hb) level and operative time were not significantly different (p>0.05).

**Platelet Response: **The platelet response to splenectomy was monitored via regular monitoring of platelet counts after surgery (table 4). In general terms, the platelet counts increased promptly and remarkably in most patients and a steady elevation was observed within the first postoperative days. In the first postoperative day, Plt count for most patients increased to above 30 × 10^9^/L. 

The platelet count response in group I was impressively lower than groups II and III (p<0.05). At discharge, 36 (60.00%) patients had complete response (> 100×10^9^/L) and 16 (26.67%) patients had Plt count of 30-100×10^9^/L, while the remaining eight (13.33%) patients had no response (Plt<30×10^9^/L). The response rate in group I was significantly lower than the other groups (p<0.001). Besides, our comparison of the treatment response based on preoperative Hb level showed that patients with higher level of Hb, achieved complete response than the other two groups (P=0.026), (table 5). The patients with no blood transfusion, had more complete response compared to patients with blood transfusion (P=0.011) (table 6).

**Table 1:** Patients’ Characteristics (frequency (percent) or mean ± SD)

		**Group I** Plt*≤10× 109 **/L**	**Group II** **10× 109<Plt≤30× 109** **/L**	**Group III 30× 109** **/L** **<Plt**	**P value**
Patients' frequency (%)		13 (21.67%)	17 (28.33%)	30 (50.00%)	0.019
Sex frequency (column %)	Male	6 (46.15%)	6 (35.29%)	12 (40.00%)	0.887
	Female	7 (53.85%)	11 (64.71%)	18 (60.00%)	
Age (y)		33.15±10.85	36.29±14.62	32.9±17.09	0.751
PRBC transfusion before surgery (mean number of PRBC**transfused)		6.00±5.66	3.33±1.53	2.00±1.23	0.265
Hemoglobin before surgery (g/dL)		10.62±2.43	11.84±3.11	11.60±3.08	0.502

^*^Platelet,

^**^Packed Red Blood Cell

**Table 2:** Operative Outcome (mean ± SD for Plt* category in day of surgery)

	**Group I Plt≤10× 10** ^9^ **/L**	**Group II** **10× 10** ^9^ **<Plt≤30× 10** ^9^ **/L**	**Group III** **30× 10** ^9^ **/L** **<Plt**	**P value**
Operative time (hour)	2.38±0.77	2.24±0.66	2.47±0.86	0.630
PRBC transfusion after surgery (mean)	3.60±1.14	3.00±2.83	3.00±1.41	0.873
Postoperative hospital stay (day)	5.00±4.10	3.65±1.17	3.27±1.41	0.070

^ *^Platelet, ^**^Packed Red Blood Cell

**Table 3:** Comparison of clinical characteristics between patients with or without blood transfusion

		**No Transfusion** **(52, 86.67%)**	**Transfusion** **(8, 13.33%)**	**P value**
Age (year)		33.23±15.07	38.38±15.45	0.157
Sex frequency (column %)	Male	21 (40.38%)	3 (37.50%)	0.999
	Female	31 (59.62%)	5 (62.50%)	
Plt^*^ in the day of surgery (x10^9^)		50.33±43.49	12.5±11.86	0.003
Hospital stay (day)		3.19±1.07	7.38±4.31	0.001
Operative time (hour)		2.35±0.78	2.63±0.74	0.366
Plt before surgery (x10^9^)		84.50±74.95	17.00±12.15	0.023
Plt After surgery (x10^9^)		135.25±88.37	56.13±63.69	0.006
Hemoglobin level before surgery (g/dL)		11.69±2.97	9.89±2.42	0.071

^ *^Platelet

**Table 4:** Platelet response to splenectomy

		**Group I** **Plt** ^*^ **≤10× 10** ^9^ **/L**	**Group II** **10× 10** ^9^ **<Plt≤30× 10** ^9^ **/L**	**Group III** **30× 10** ^9^ **/L** **<Plt**	**P value**
Platelet count after surgery × 10^3^		50.31±55.26	117.35±73.22	161.10±90.03	<0.001
Response rate, frequency (% within groups)	CR^**^ 36 (60.00%)	2 (15.38%)	9 (52.94%)	25 (83.33%)	<0.001
	R^***^16 (26.67%)	4 (30.77%)	7 (41.18%)	5 (16.67%)	
	NR^****^8(13.33%)	7 (53.85%)	1 (5.88%)	0 (0%)	

^*^Platelet, ^**^CR: Complete Response (100 × 10^9^/L <Plt), ^***^R: Response (30-100 × 10^9^/L), ^****^NR: No Response (Plt≤30× 10^9^/L)

**Table 5:** Comparison of treatment response according to hemoglobin level before surgery

	**NR** ^*^ ** (I)**	**R** ^**^ ** (II)**	**CR** ^***^ ** (III)**	**P value**	**P value**		
					**I vs. II**	**II vs. III**	**I vs. III**
Hemoglobin level (g/dL)	9.63±1.38	10.58±2.19	12.25±3.23	0.026	0.717	0.126	0.052

^ *^NR: No Response (Plt≤30× 10^9^/L), ^**^R: Response (30-100 × 10^9^/L), ^***^CR: Complete Response (100 × 10^9^/L <Plt)

**Table 6:** Comparison of treatment response between different groups of recipients of packed red blood cell after surgery

		**Not Transfused (n=52)**	**Transfused** **(n=8)**	**Total**	**P value**
Response rate, n (%)	CR^*^	34 (56.7%)	2 (3.3%)	36 (60.0%)	0.011
	R^**^	14 (23.3%)	2 (3.3%)	16 (26.7%)	
	NR^***^	4 (6.7%)	4 (6.7%)	8 (13.3%)	

^ *^CR: Complete Response (100 × 10^9^/L <Plt), ^**^R: Response (30-100 × 10^9^/L), ^***^NR: No Response (Plt≤30× 10^9^/L)

## Discussion

Idiopathic thrombocytopenic purpura is the prevalent hematologic disorder for all ages, with incidence of more than 2 in 100000 annually and remission rate of 5-11% in adults (20). According to a large series, splenectomy required in 20-45% patients but the exact rate of necessity to splenectomy remains unclear (1, 3, 5). Based on the studies, after splenectomy, the remission rate in adult and relapse occurred approximately 88% and 15%, respectively. According to our data, 36 (60%) patients had complete response, 16 (26.7%) patients showed a response and 8 (13.3%) patients had no response to splenectomy (table 6). Laparoscopic splenectomy is an accepted procedure and well established modality in ITP patients, and appears to be safe, efficient and the best option in patients resistant to glucocorticoid therapy, who are candidates for elective splenectomy (21, 22). Furthermore, some authors recommended early splenectomy in ITP patients with long history of glucocorticoid therapy and rapid response to medical treatment to decrease the duration and indeed the morbidity of medical therapy (1,3, 23).

Despite the advantages of laparoscopic splenectomy in ITP patients, the safe level of preoperative Hb and platelet count, their impact on postoperative results and necessity for transfusion during or after the procedure remain unclear. Based on this study, patients with higher Hb level and platelet count >30 × 10^9^/L showed complete response but patients who need PRBC transfusion and platelet<10× 10^9^/L showed no response. The eventual contest among surgeons can be attributed to the major risk of preoperative bleeding because of low Plt count. In ITP patients, as the Plt count decreases, the mean Plt volume increases, furthermore the larger platelet seems to be younger and more reactive (24, 25). So the bleeding time of these patients is usually shorter than expected based on the level of thrombocytopenia, and actually the severity of bleeding is less than the situation which the thrombocytopenia is due to bone marrow failure (26).

According to the British Committee for Standards in Hematology (BCSH) guidelines, the mean Plt count recommended for splenectomy is 30 × 10^3^ (7). BCSH guidelines recommend glucocorticoids and/or IV Ig in the treatment of affected patients but a majority of them never achieve a satisfactory promotion. Prophylactic perioperative transfusion of Plt is considered for patients with Plt count < 10 × 10^9^/L once the splenic artery is ligated, but there is not any accepted consensus on the appropriate time for transfusion of Plt and the exact efficacy of it in ITP patients. Some studies declared that laparoscopic splenectomy can be done safely in patients with a platelet count lower than 10×10^9^/L without any platelet transfusion if there is no evidence of preoperative severe coagulopathy or bleeding (27-31). 

In candidates for laparoscopic splenectomy, the first important issue is to provide a safe anesthesia. Airway bleeding caused by intubation, trauma and intracranial hemorrhage caused by coughing after introduction of tracheal tube may result in major morbidity and mortality (30). In the ITP patients, preoperative transfusion of Plt cannot result in a satisfactory promotion in Plt level, therefore, in this situation, transfusion of PRBC may limit the complications of hemorrhage. The other concerning problem is bleeding due to surgical trauma. Actually, these patients are more subject to intraoperative bleeding, leading to higher complications and greater requirements to blood transfusion (14, 31). In our study, the patients with a very low platelet count needed for more blood transfusion and relatively had more intraoperative blood loss, and therefore received more PRBC, but there was not any significant difference between the patients according to the Plt level. Interestingly, the mean Plt count before surgery, in the day of surgery and after surgery, and remission rate in non-transfused patients was significantly higher than patients who needed transfusion.

Although, in the past decades, it has been accepted to administer PRBC transfusion to severely thrombocytopenic individuals but there is no evidence to confirm preoperative PRBC transfusion has any efficacy in patients who are indicated for laparoscopic splenectomy (27). Accordingly, in this study, the Hb level was not significantly different in these two groups. After all, blood transfusion did not cause any significant effect on the consequences of this procedure.

Based on the electron microscopic studies, thrombocytopenia is associated with step by step (piecemeal) thinning of the vessel wall endothelium due to development of gaps between endothelium cells which may result in extravasation of red blood cells (27). This condition can lead to mild to severe anemia in ITP patients with very low platelet counts before surgery relatively because of mucocutaneous bleedings. Blood transfusion can decrease the risk of anemia in these patients. However the recent advances in surgical techniques like laparoscopic splenectomy significantly lowered the necessity for Plt and blood transfusion in comparison to open surgery methods (32, 33). 

In conclusions, laparoscopic splenectomy in patients with chronic idiopathic thrombocytopenic purpura and very low platelet count is a safe and effective procedure with low complications. Although in some ITP patients, blood transfusion is considered as a routine part of orders, our experience showed that very low Plt count (< 10×10^9^/L) with a normal coagulation profile is not contraindicated for laparoscopic splenectomy, do not increase complications and blood transfusion may not be necessary during surgery. But further investigations are needed to make clear the exact role of blood transfusion in these patients. Based on our results, it seems that intraoperative bleeding can be greatly prevented by increased skills, precise and meticulous methods, adequate instrumentation and advanced techniques of laparoscopic splenectomy. 
